# Quantitative Segmentation of Fluorescence Microscopy Images of Heterogeneous Tissue: Application to the Detection of Residual Disease in Tumor Margins

**DOI:** 10.1371/journal.pone.0066198

**Published:** 2013-06-18

**Authors:** Jenna L. Mueller, Zachary T. Harmany, Jeffrey K. Mito, Stephanie A. Kennedy, Yongbaek Kim, Leslie Dodd, Joseph Geradts, David G. Kirsch, Rebecca M. Willett, J. Quincy Brown, Nimmi Ramanujam

**Affiliations:** 1 Department of Biomedical Engineering, Duke University, Durham, North Carolina, United States of America; 2 Department of Electrical and Computer Engineering, Duke University, Durham, North Carolina, United States of America; 3 Department of Pharmacology & Cancer Biology, Duke University School of Medicine, Durham, North Carolina, United States of America; 4 Laboratory of Veterinary Clinical Pathology, College of Veterinary Medicine, Seoul National University, Seoul, South Korea; 5 Department of Pathology, University of North Carolina School of Medicine, Chapel Hill, North Carolina, United States of America; 6 Department of Pathology, Duke University Medical Center, Durham, North Carolina, United States of America; 7 Department of Radiation Oncology, Duke University School of Medicine, Durham, North Carolina, United States of America; 8 Department of Biomedical Engineering, Tulane University, New Orleans, Louisiana, United States of America; University of Navarra, Spain

## Abstract

**Purpose:**

To develop a robust tool for quantitative *in situ* pathology that allows visualization of heterogeneous tissue morphology and segmentation and quantification of image features.

**Materials and Methods:**

Tissue excised from a genetically engineered mouse model of sarcoma was imaged using a subcellular resolution microendoscope after topical application of a fluorescent anatomical contrast agent: acriflavine. An algorithm based on sparse component analysis (SCA) and the circle transform (CT) was developed for image segmentation and quantification of distinct tissue types. The accuracy of our approach was quantified through simulations of tumor and muscle images. Specifically, tumor, muscle, and tumor+muscle tissue images were simulated because these tissue types were most commonly observed in sarcoma margins. Simulations were based on tissue characteristics observed in pathology slides. The potential clinical utility of our approach was evaluated by imaging excised margins and the tumor bed in a cohort of mice after surgical resection of sarcoma.

**Results:**

Simulation experiments revealed that SCA+CT achieved the lowest errors for larger nuclear sizes and for higher contrast ratios (nuclei intensity/background intensity). For imaging of tumor margins, SCA+CT effectively isolated nuclei from tumor, muscle, adipose, and tumor+muscle tissue types. Differences in density were correctly identified with SCA+CT in a cohort of *ex vivo* and *in vivo* images, thus illustrating the diagnostic potential of our approach.

**Conclusion:**

The combination of a subcellular-resolution microendoscope, acriflavine staining, and SCA+CT can be used to accurately isolate nuclei and quantify their density in anatomical images of heterogeneous tissue.

## Introduction

Optical microscopy is a powerful technique to obtain high-resolution images of tissue histology in real-time at the point-of-care, without the need for fixing, sectioning, and staining. Various optical microscopy techniques including reflectance and fluorescence [1]–[3], Raman [4], confocal [5], [6], and optical coherence tomography [7], [8] have been used to exploit intrinsic sources of contrast in thick tissues. Additionally, fluorescence microscopy has been combined with vital fluorescent stains such as acridine orange (AO) [9]–[11], acriflavine [12], [13], and DAPI [14] to visualize micro-anatomical features in skin [9], breast [14], ovarian [11], oral [12], and esophageal [13] cancers. All of these technologies enable rapid and completely non-destructive visualization of tissue histology.

Robust methods for segmentation and quantitative analysis are essential to enable automated and rapid surveillance of tissue pathology, particularly when images are collected in near real time. There are three important criteria that have to be considered in the selection of an appropriate image analysis strategy. (1) If the background patterns and intensities vary greatly between images (i.e. if images are heterogeneous), will a method still be able to isolate features of interest, such as tumor nuclei? (2) Can the method resolve overlapping nuclei when attempting to characterize nuclear size or density? (3) Does the method require human intervention and supervision, thus introducing subjective bias and complexity into the analysis?

Many approaches for cell or cell nuclei segmentation exist. A summary of the advantages and disadvantages of commonly used approaches for nuclei segmentation in microscopy is included in Table 1. Global thresholding approaches work well when cell nuclei do not overlap and background intensities are evenly distributed, and its use in isolating cell nuclei is well established in the literature [11], [12], [14], [15]. However, it is also broadly recognized that global thresholding has many shortcomings, specifically that it has limited utility in heterogeneous images in which background intensities vary greatly. While global thresholding takes intensity information into account, it does not incorporate *geometric* information, such as the expected size or shape of nuclei. Thus, in an effort to take geometry into account, many groups have developed techniques that combine global and local image information, such as adaptive window thresholding or local maxima detection [15]–[21], active contours [22]–[25], watershed segmentation [21], [26], [27], high pass filtering [28], and the circle transform [29]. In adaptive window thresholding or local maxima detection, regions or windows of the image are examined separately and the nuclei within each region are identified based on intensity information through either finding maximum intensities or applying a threshold [15]–[21]. For heterogeneous images, the window size and threshold within each window should ideally vary across images and patients in order to effectively segment nuclei which are surrounded by various structures, such as muscle, adipose tissue, or other types of connective tissue. Tuning so many parameters on an image by image basis quickly can become unmanageable and introduce subjective bias into the quantification of nuclear size and density. Active contours, such as snakes, find the boundary of a feature by minimizing an “energy” function associated with the current contour that measures the contour’s curvature and enclosed area [22]–[25]. However, choosing or defining the energy function can be a complex process, and segmentation results are highly sensitive to this choice. Additionally, active contours require human intervention and supervision through manually guiding the outlining of features or selecting a pixel in the interior of each structure (e.g. tumor nucleus) to be extracted. In images that contain large collections of nuclei, this segmentation approach can quickly become unwieldy. Furthermore, due to the complexity of this computational technique, it is difficult to know when an optimal solution has been achieved. In watershed segmentation methods, an image is partitioned into regions separated by watershed lines. While watershed segmentation can identify overlapping nuclei, it is vulnerable to a well-recognized phenomenon called over-segmentation, in which homogeneous regions are segmented into multiple different regions erroneously [21], [26], [27]. These effects can be somewhat mitigated via an involved parameter tuning process requiring significant human intervention or through variations to the watershed transform, such as viscous watershed [30]; generally, this is an area of active ongoing research. Furthermore, there are many regions in an image that are segmented via watershed methods that are not meaningful for our purpose of isolating nuclei within heterogeneous images. For example in heterogeneous images, the improper segmentation of background elements such as adipose or connective tissue can mistakenly be identified as nuclei, leading to incorrect quantitation of nuclear size and density. High pass filtering is a technique that is commonly used to isolate edges in images, and can be used to segment small features such as nuclei [28]. While high pass filtering is simple to implement and easy to tune, it is highly sensitive to noise present in an image. Lastly, the circle transform can be used to detect approximately circular objects of a specified range of radii within an image [29]. While this technique is simple and can identify overlapping nuclei, it assumes that objects are approximately circular and is sensitive to small variation in background intensity. Because of this sensitivity to small variations in the background, the circle transform, in isolation, is a suboptimal approach for heterogeneous images.

**Table 1:** Nuclei segmentation methods.

Method	Advantages	Disadvantages
Global thresholding	Simple, easy to tune	Requires uniform background intensity
Adaptive thresholding	Simple	Requires varying window size across image and adjusting threshold within each window
Active contours	Can find object outlines in complex images	Requires defining complex energy function and human intervention and supervision
Watershed segmentation	Can identify overlapping nuclei	Results in over segmentation
High pass filter	Simple, easy to tune	Sensitive to noise
Circle transform	Simple, can identify overlapping nuclei	Sensitive to small variations in background intensity

Despite the diversity of approaches, segmentation of cells and cell nuclei remains a challenge due to the complexity of images that have varying levels of contrast and non-uniform background heterogeneity, as well as overlapping nuclear features. To address this important need, a computational technique is described here that leverages morphologic information inherent in monochrome images of fluorescently-stained microanatomy to separate and quantify the presence of distinct tissue types in a heterogeneous image. In combination with fluorescence microendoscopy and the contrast agent, acriflavine [31], [32] the utility of this technique in the visualization of tissue histology in tumor margins is demonstrated. More specifically, the model-based approach decomposes tissue histology images into mathematically discrete components. First, sparse component analysis (SCA) [33] is used to separate cell nuclei, fibrous components, and adipose components. Second, the circle transform (CT) [29] is applied to the deconstructed image to quantify the size and density of overlapping features of interest, in this case, nuclei as a means to identify the presence of residual disease in a tumor margin. While the CT is sensitive to small variation in the background, this effect is mitigated by first using SCA to remove the background. This manuscript describes a methodology that systematically evaluates the potential of an image processing approach or combination of approaches, for a specific biomedical problem. The image processing approach chosen here is SCA followed by the CT, and the specific indication is the ability to isolate nuclei from heterogeneous tumor margin images. The rationale for selecting SCA is that it can segment different types of structures (nuclei, muscle, and adipose tissue) in complex heterogeneous images. The CT was chosen to isolate nuclei because it can distinguish overlapping circular nuclei and is easy to tune. It should be noted that the combination of SCA+CT is not the only solution to this complex problem; however, it is a well-justified approach to analyzing images from heterogeneous tissues and certainly could be adapted to include other methods if they can benefit the overall approach. Unlike image processing techniques which rely solely on intensity information (and are thus susceptible to calibration errors), SCA incorporates geometric information through the property of sparsity. This leads to a highly flexible approach that requires tuning a very small number of parameters, can resolve overlapping nuclei, and does not require human intervention or supervision. Additionally, this technique does not discard image content but rather retains all of the image information inherent in the image to preserve spatial relationships between tissue types, which are essential for proper interpretation of the images. Any clinical application in which one needs to assess the pathological state of disease at the point of care (during a procedure) could benefit from this combination of approaches [34]–[36].

Our purpose is to demonstrate that SCA+CT can accurately isolate and quantify information within an image with a single stain both on the excised margin and more importantly, on the intact tumor bed. The sensitivity of the SCA+CT approach to variations in nuclear size, density and background heterogeneity is demonstrated through simulations. The quantitative attributes of this strategy are demonstrated by comparing the output of monochrome fluorescence images of tissue sections to that derived from histology. The clinical utility of this approach to detect residual disease is examined by imaging excised margins as well as the tumor bed *in vivo* in a cohort of mice after surgical resection of a sarcoma [37], [38].

## Materials and Methods

### Ethics Statement

This study was carried out in strict accordance with the recommendations in the Guide for the Care and Use of Laboratory Animals of the National Institutes of Health. The protocol was approved by the Duke University Institutional Animal Care and Use Committee (Protocol Number: A134-10-05). All surgery was performed under isoflurane gas anesthesia, and all efforts were made to minimize suffering.

### Mice and Sarcoma Generation

The generation of the temporally- and spatially-restricted genetically engineered mouse model of sarcoma was performed as described by Kirsch et al [37]. Briefly, mice were anesthetized using isoflurane and soft tissue sarcomas were generated by intramuscular injection of a calcium phosphate precipitate of Ad-Cre (Gene Transfer Vector Core, University of Iowa) in to the proximal portion of the medial or lateral gastrocnemius muscle [37]. Mice were on a mixed 129 SvJae/C57/Bl6 background for these studies. Tumors were excised as described by Mito et al [34]. Mouse genotypes used to generate sarcomas included *LSL-Kras^G12D/+^*;*Trp53^Flox/Flox^* [37] and *Braf ^Ca/+^;Trp53 ^Flox/Flox^* [39].

### Imaging System and Contrast Agent

A fluorescence microendoscope device that has previously been described in detail [12] was used to collect images of acriflavine stained tissues. The system contained a 455 nm light emitting diode (Luxeon V Star, LXHL-LR5C), excitation filter (Semrock, FF01-452/45-25) dichroic mirror (Chroma 485 DCLP), emission filter (Semrock, FF01-550/88-25), CCD camera (Point Grey Research, GRAS-14S5), and coherent fiber bundle (Sumitomo, IGN-08/30). The fiber bundle was composed of 30,000 fibers giving a circular field of view of approximately 750 µm in diameter. The resolution of the system was 4.4 µm. For both *ex vivo* and *in vivo* studies, images were produced by placing the fiber bundle in contact with the acriflavine stained tissue surface. Acriflavine (0.01% w/v, Sigma-Aldrich) dissolved in phosphate buffered saline (PBS) was topically applied to all *ex vivo* and *in vivo* specimens immediately prior to imaging.

### *Ex vivo* Imaging of Excised Tissue Margins

Seven mice were euthanized immediately prior to surgical tumor resection. Within ten minutes of euthanasia, the tumor was excised from the leg. Seven tumors were excised, six of which were imaged directly (bulk tissue imaging) and one of which was used for serial tissue sectioning (tissue section imaging).

For tissue section imaging, the excised tissue was flash frozen in liquid nitrogen, imbedded in optimal cutting temperature compound (Tissue-Tek), serially sectioned, and mounted on glass slides. Alternating 50 µm and 5 µm sections were cut with a Leica cryostat with 1–2 sections discarded between to allow for cryostat adjustment. 3–5 drops (0.15–0.25 mLs) of acriflavine was topically applied to the 50 µm sections, and after 30 seconds, the tissue sections were raster-scanned with the fiber probe in 1 mm increments to create mosaics. The alternating 5 µm sections were submitted for standard hematoxylin and eosin (H&E) staining.

For bulk tissue imaging, the tissue was laid flat and 3–5 drops (0.15–0.25 mLs) of acriflavine were topically applied. 30 seconds following the application of acriflavine, the distal end of the fiber bundle was placed in contact with the tissue and images were acquired from several discrete sites on the tissue (3 to 5 sites per specimen). Each imaged site was inked with a 1 mm dot to facilitate pathologic co-registration. The tissue was fixed, paraffin-embedded and sectioned. *En face* sections of inked regions were taken below the inked surface. The H&E stained slides were reviewed separately by three pathologists. For each H&E slide, the tissue was diagnosed as tumor, muscle, adipose, or any combination thereof. A total of eight tumor images, thirteen muscle images, six tumor+muscle images, and one adipose image were acquired from six animals.

### *In vivo* Imaging of the Resected Tumor Cavity

For the *in vivo* study, surgeries were performed under isoflurane gas anesthesia. Buprenorphine and Bupivacaine were delivered for peri-operative analgesia. A total of two animals were included in the *in vivo* study. After induction with isoflurane anesthesia and analgesic treatment, the grossly apparent soft tissue sarcoma was surgically removed. Post-resection, 3–5 drops (0.15–0.25 mLs) of 0.01% (w/v) acriflavine dissolved in PBS was topically applied to the resection cavity. 30 seconds following the application of acriflavine, the distal end of the fiber bundle was placed in contact with the tissue and images were acquired. The tumor bed was raster scanned to create mosaics of the margin by systematically moving the probe in 1 mm increments across the entire tumor bed. Next, the surface of the excised specimen that mirrored the tumor bed was inked to orient the specimen and facilitate pathological assessment of the resected margin. The most superficial section was submitted for H&E processing and evaluation by three pathologists. If the section contained tumor, the margin was diagnosed as pathologically positive. If the section did not contain tumor, the margin was considered pathologically negative. Additionally, the mice were monitored for local recurrence at the excision site for up to 120 days.

### Generation of Simulated Sarcoma Images

Tumor, muscle, and tumor+muscle tissue images were simulated because these tissue types were most commonly observed in sarcoma margins. All simulated nuclei were drawn the same way using MATLAB (2009a, Mathworks Inc., Natick, MA). Specifically, random locations were selected within each region. Then the fspecial command was used to create a disk of a specified radius at each location. The disk was then blurred with a Gaussian filter that had a standard deviation of 1.1 pixels. The blurring step was done to simulate the gradual falloff in intensity seen in nuclei imaged experimentally. Muscle was simulated as longitudinal fibers, and tumor+muscle was simulated as tumor nuclei on top of the longitudinal fibers, which was the weighted addition of the two components. Specifically, for the tumor+muscle simulations the nuclei phantom was multiplied by a weighting factor and added to the muscle phantom. The ratios between tumor nuclei and the underlying muscle were chosen based on observations from experimental images and were varied from 1.2–1.8. The contrast ratios represent the maximum nuclei intensity divided by the maximum muscle intensity. See methods S1 for more details.

### Sparse Component Analysis and Circle Transform (SCA+CT)

All processing of the images was performed using MATLAB (2009a, Mathworks Inc., Natick, MA). Images were preprocessed to remove the rim of the fiber bundle and the fiber core pattern superimposed onto each image. For more details see methods S1 and **Fig. S1**. Tissue components (nuclei, muscle fibers, and the outline of adipose cells) were separated computationally using a sparse component analysis (SCA) method. Let *y* denote the preprocessed image data, modeled as
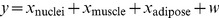
(1)where 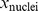, 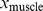, and 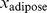 denote the true nuclei, muscle, and adipose components respectively, and *w* accounts for noise and small deviations from the model. The key assumption was that each tissue component has a different “sparsifying” basis or dictionary in which the expansion coefficients were nearly all zero, with only a few large coefficients. (For instance, an image of muscle fibers was relatively smooth, so it could be accurately approximated using a superposition of a small number of Fourier basis functions.) If the sparsifying dictionaries were sufficiently dissimilar, then the sparsity could be exploited to uniquely identify the different tissue components.

The pixel basis was used for the nuclei dictionary to capture the small and spatially isolated nuclei. The discrete cosine transform (DCT), a variant of the Fourier transform, basis was used to describe muscle components with periodic fiber structures. Specifically the DCT was performed on the entire image (not in blocks) to capture globally smooth muscle features. Mathematically, 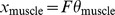, where *F* was a matrix representation of the DCT, and 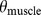 was a vector of the DCT coefficients; most elements of 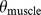 were zero. The curvelet dictionary was used to represent the curvilinear outlines of adipose cells [40]. Adipose tissue can be described as localized piecewise smooth features, and therefore curvelets are well suited to capture adipose features. Specifically, curvelets, which are similar to wavelets, have dictionary elements corresponding to different scales and locations throughout an image and is relatively dissimilar to both the pixel and DCT bases [40]. Let *C* denote the curvelet transform matrix so that 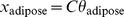, where 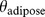 was a sparse curvelet coefficient vector.

The sparse coefficient vectors were estimated by solving a regularized least-squares inversion [33]:
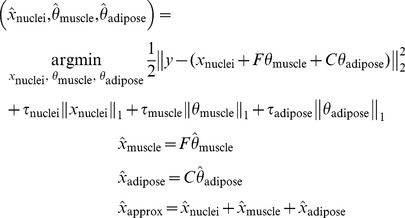
(2)where 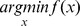 returned the value of *x* (the argument) that minimized *f,* 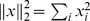 (the 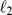 norm) and 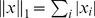 (the 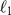 norm), where 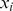 was the *i* ^th^ component of *x*. The 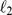 term in Eqn. 2 ensured that the approximation 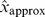 was a good fit to the observed data, while the 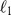 terms promoted sparsity in the variables 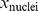, 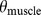, and 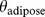. The regularization parameters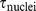, 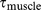, and 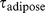 were positive weights that controlled the balance between data fidelity and sparsity in the reconstruction. These parameters were selected using an empirical method described in methods S1. To solve this minimization problem, the Gradient Projection for Sparse Reconstruction (GPSR) algorithm was used [41]. After SCA was applied to isolate nuclei, the nuclear size and density were quantified by computing the circle transform [29] on 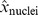 to detect approximately circular objects (*i.e.*, nuclei). This methodology, which is referred to as SCA+CT, was applied to analyze all images in this study. Example images and code are available at http://hdl.handle.net/10161/6378.

### Statistical Analysis

Wilcoxon rank sums (non-parametric, two-tailed, alpha = 0.95) were used to determine whether quantitative image parameters were significantly different between tumor and muscle and between tumor+muscle and muscle tissue types for the bulk tissue image set. A significance level of *p*<0.05 was considered to reject the null hypothesis for all analyses. The *n* for each statistical analysis is listed in the respective figure legend. Receiver operating characteristic (ROC) curves and the area under the curve (AUC) were calculated for nuclear density using a web-based tool [42]. The Youden index, which is a frequently used summary measure for ROC curves, was calculated for each ROC curve, and the associated sensitivity and specificity is reported [43].

## Results

### Illustration of Challenges Associated with Nuclei Segmentation in Heterogeneous Tissue

**Figure 1:**
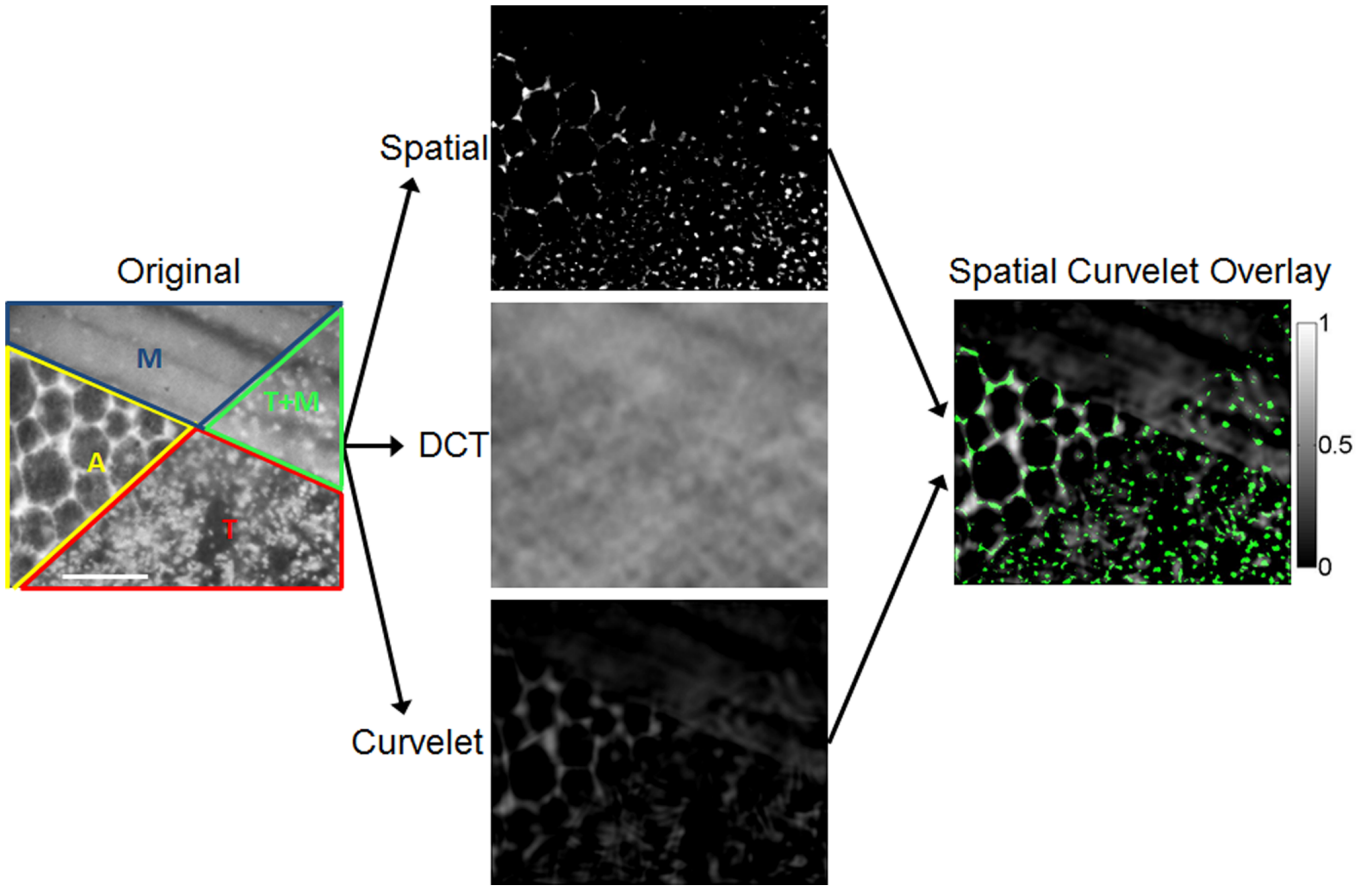
Illustration of challenges associated with nuclei segmentation in heterogeneous tissue. The original image was created by blending different regions of a tumor (T), muscle (M), adipose (A), and tumor+muscle (T+M) image together. This image was put through SCA to yield the spatial, DCT, and curvelet outputs. Then, the spatial image was false colored green and laid on top of the curvelet image to yield to spatial curvelet overlay. This illustrates how the nuclei are spatially co-registered with some of the features isolated in the curvelet output. This information could be used diagnostically to indicate that these nuclei are associated with benign adipose tissue, not with malignant tissue.

First to illustrate the challenges associated with isolating nuclei in heterogeneous tissue, an image was created with four different tissue types present, including tumor, muscle, adipose, and tumor+muscle tissues. Due to the small field of view of the fluorescence microendoscope (0.63 mm^2^) it was not possible to capture all four tissue types in a single image. Therefore, in order to illuminate the challenges associated with isolating nuclei from different tissue types, the original image shown in **Fig. 1** was created by blending different regions of a tumor, muscle, adipose, and tumor+muscle image together. As can be seen, there are inherent challenges in segmenting nuclei from each of these regions with a single approach. Specifically, the goal is to isolate the large amount of nuclei from the tumor and tumor+muscle regions without capturing any of the background and to isolate the nuclei from the adipose region that are located on the edges of the adipocytes. To illustrate the ability of SCA to meet these goals, the original image was put through SCA to yield the spatial, DCT, and curvelet outputs. From the spatial output we can see that nuclei are correctly isolated throughout the tumor and tumor+muscle regions, as well as in the adipose region. Lastly, SCA does not discard image content but rather retains all of the image information inherent in the image. For example, the curvilinear structures present in the original image, particularly in the adipose region, are isolated in the curvelet output. This information can be used to properly interpret images. To illustrate this point the nuclei isolated in the spatial output were false colored green and then overlaid onto the curvelet output. As can be seen, the nuclei in the adipose region are spatially co-registered with the outline of the adipocytes. This information could be used diagnostically to indicate that these nuclei are associated with benign adipose tissue, not with malignant tissue.

### Sensitivity of SCA+CT to Background Heterogeneity, Nuclear Size and Density

**Figure 2:**
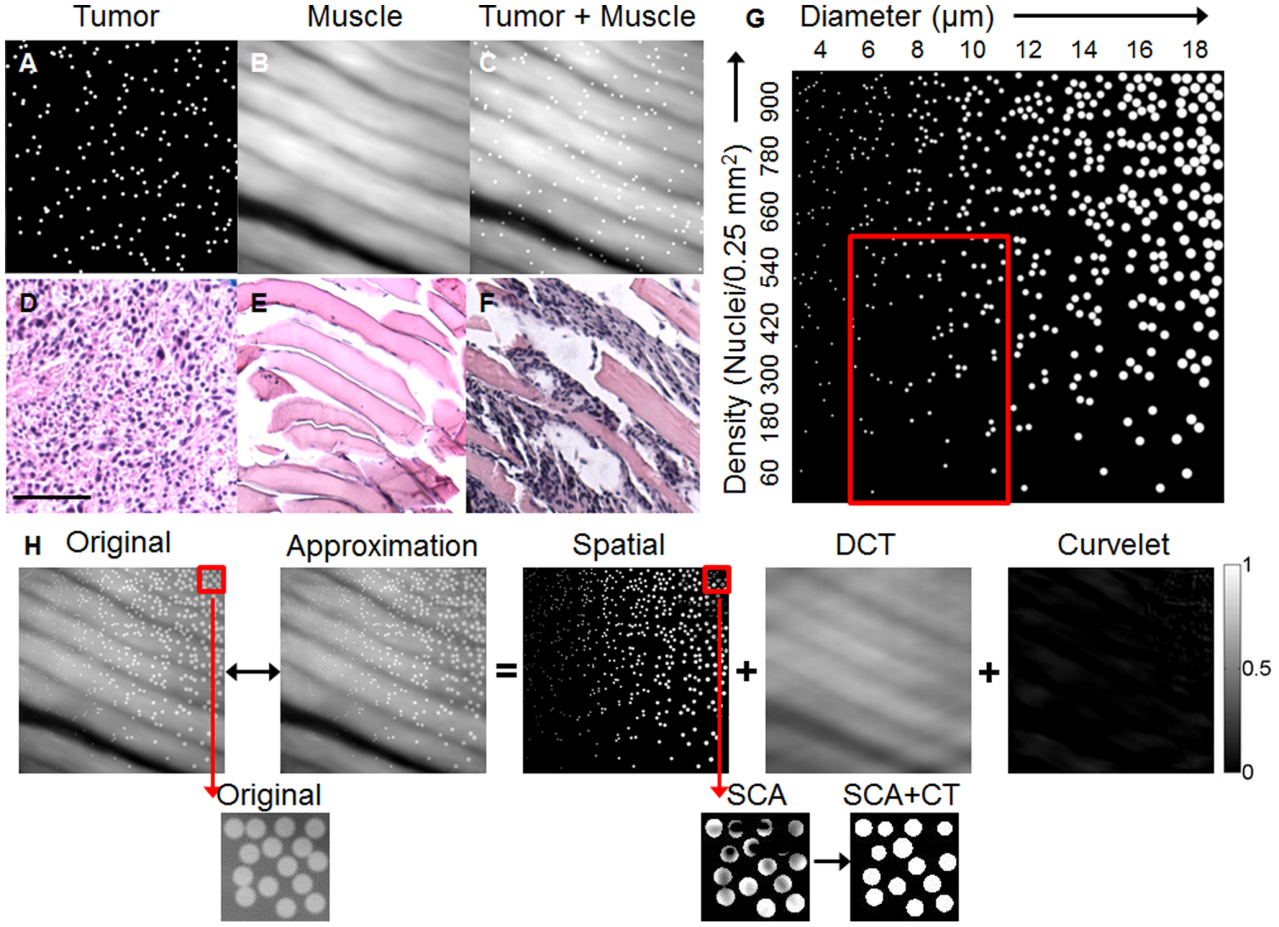
Image simulations used to evaluate SCA. Simulations of tumor (**a**), muscle (**b**), and tumor+muscle (**c**) were mimicked after observations seen in H&E sections (**d–f**). (**g**) To better understand how well SCA can capture nuclei of various sizes and densities, a single simulated image was created that contained a variety of sizes and densities. The red box indicates the most commonly seen nuclear sizes and densities in our data. The outputs of SCA for a tumor+muscle simulation are shown (**h**). The colorbar shows the gray level intensities, which vary from 0 to 1. In this case, the curvelet image appears blank because no adipose is present in the original image. A zoomed in area of the upper right hand corner of the original image is shown. The same area in the spatial image is shown with CT applied to yield SCA+CT. Scale bar 50 µm.

In order to determine the accuracy and sensitivity of SCA+CT to quantify nuclear size and density, a series of simulated images were generated in which the size and density of nuclei could be varied in the presence of background tissue heterogeneity. Tumor was simulated as randomly dispersed circular nuclei (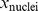, **Fig. 2a**), muscle was simulated as longitudinal fibers (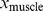, **Fig. 2b**), and tumor+muscle was simulated as tumor nuclei on top of the longitudinal fibers, which was the weighted addition of the tumor and muscle components (**Fig. 2c**). These simulations were based on characteristics observed in H&E stained histological sections of the same tissue (**Fig. 2d–f**). Specifically, nuclei were simulated with diameters ranging from 4–15 µm (4–18 pixels) and densities from 60–900 nuclei/0.25 mm^2^. These values were selected based on the biologically expected ranges. The simulated nuclei in **Fig. 2g** were added to the simulated muscle image to yield the tumor+muscle simulated image 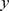 (original, **Fig. 2h**) which was the input image into SCA. The outputs of SCA include the spatial (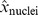), DCT (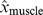), and curvelet (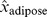) component estimates, whose summation was denoted as the approximation (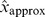) of the original image. Next, CT was applied to 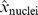 to isolate overlapping nuclei for accurate quantitation of size and density. A zoomed in area in the upper right hand corner of the spatial image (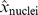) is shown with CT applied to yield SCA+CT (**Fig. 2h**). All subsequent images were analyzed with SCA+CT.

**Figure 3:**
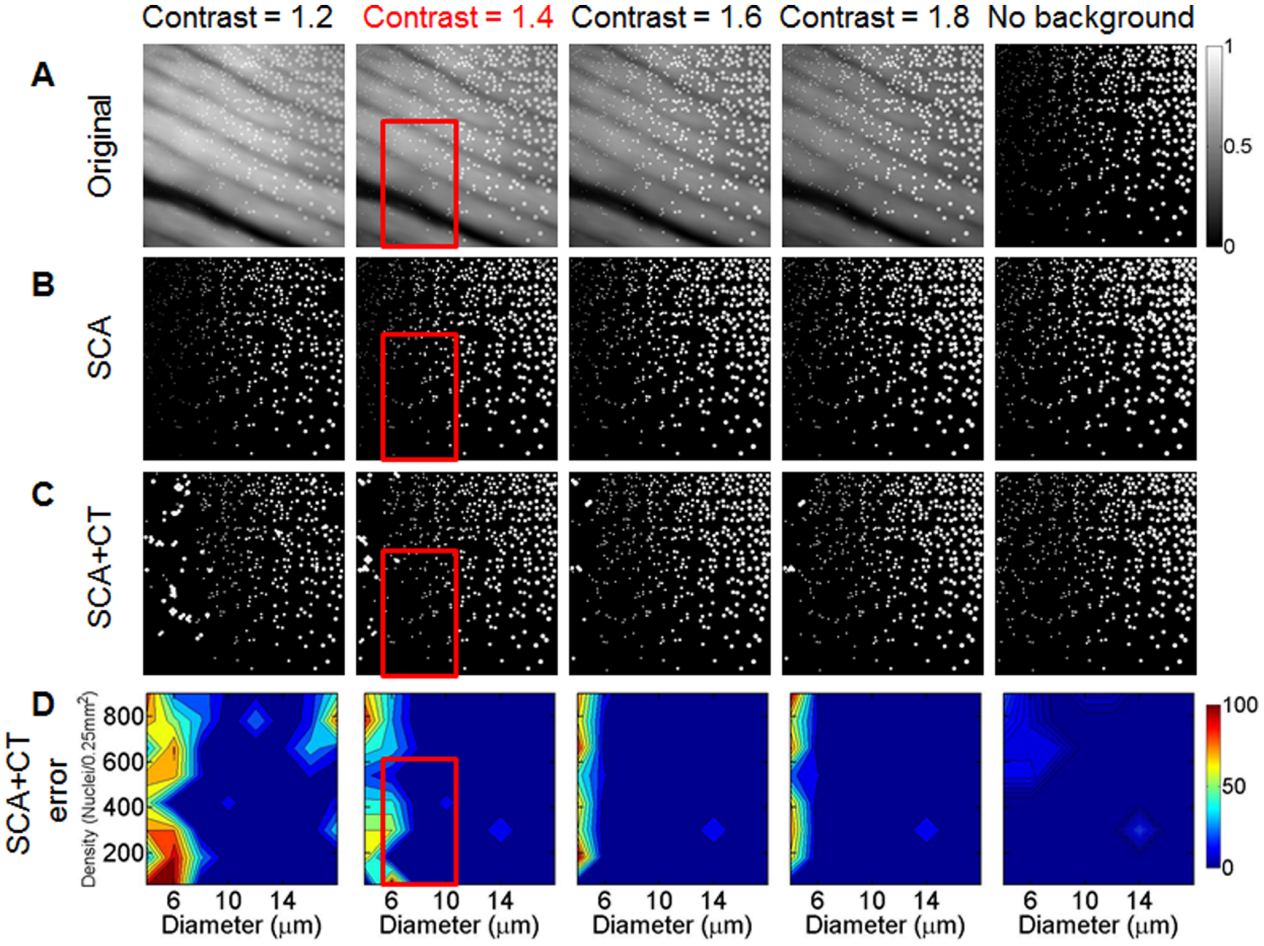
SCA performance varies with nuclear size, nuclear density, and background contrast. (**a**) The image simulation with various nuclear sizes and densities was added to the muscle simulation with varying weights or ratios to create a tumor+muscle simulation. The ratios are reported as the (max tumor nuclei intensity/max muscle intensity). The spatial output from SCA is shown in (**b**). The nuclear density was calculated by applying CT to the images in (**b**) to yield the images in (**c**). Red boxes indicate the most commonly seen nuclear size, nuclear density, and contrast ratio observed in our data. The percent error was calculated and is shown as a contour plot in (**d**). The colorbar indicates the percent error (%).

A set of simulated images with varying contrast ratios was used to examine the influence of background heterogeneity on the ability of SCA+CT to quantify nuclear size and density (**Fig. 3a**). Nuclei from each image were isolated with SCA (**Fig. 3b**) and quantified with CT (**Fig. 3c**). The percent error was calculated by comparing the densities calculated from the simulated images in **Fig. 3c** to the original densities in the tumor simulation, and can be seen in **Fig. 3d**. As seen, the overall error decreases as contrast increases. The highest errors are observed on the left hand side of each image, which corresponds to the area with the smallest nuclei. Because CT is based on local gradients, it is unable to distinguish nuclei that are smaller than 5 pixels in diameter.

### Trends Observed in Nuclear Size and Density Quantified using SCA+CT

**Figure 4:**
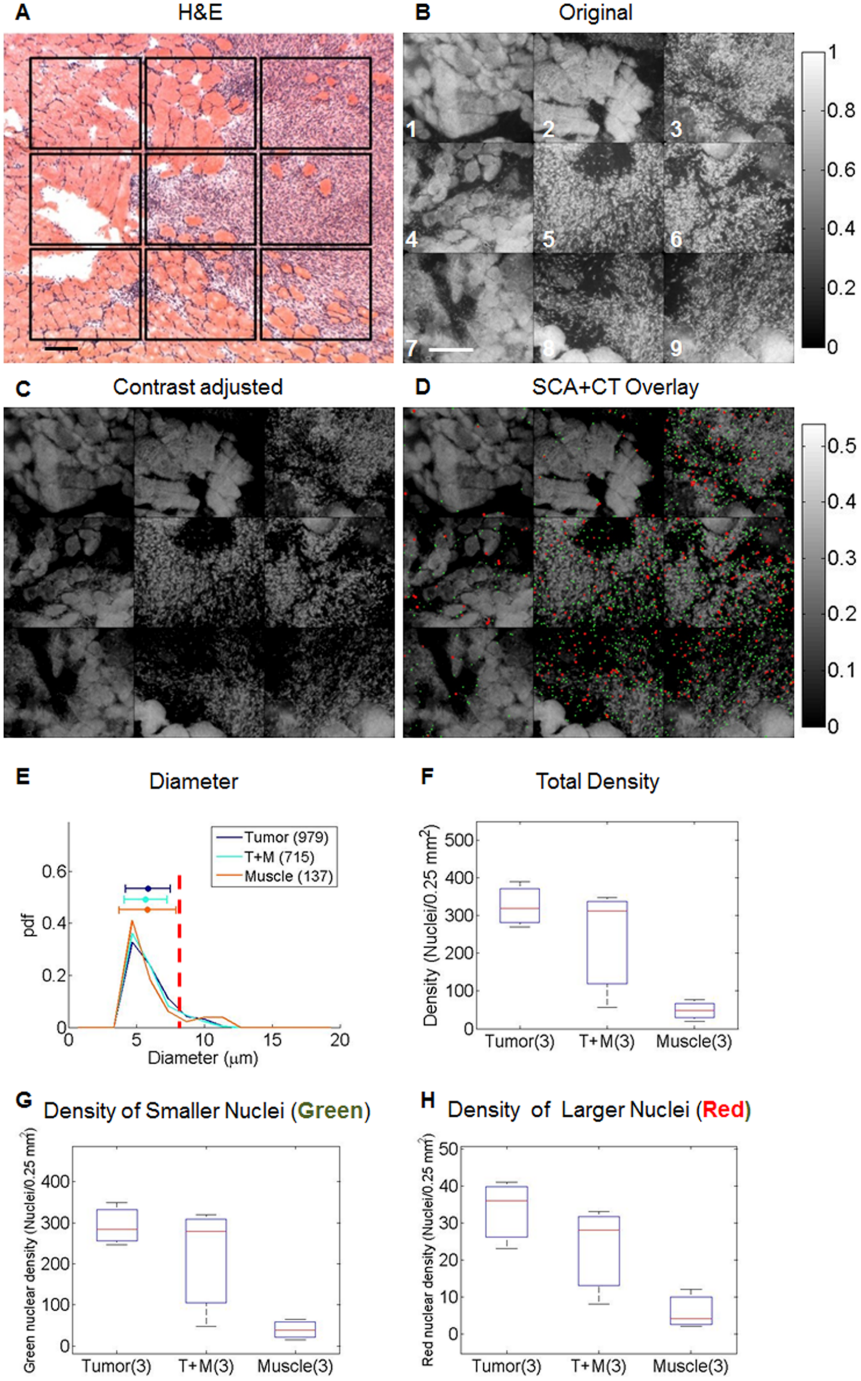
SCA+CT applied to a panel of tissue section images illustrates trends in nuclear density. The specific locations where fluorescence microendoscopy (FM) images (Original, (**b**)) were collected are indicated by squares in the H&E section (H&E, (**a**)). Nuclei were isolated and quantified by using SCA+CT, which is illustrated in (**d**). For SCA+CT, nuclei are overlaid onto the original image. Nuclei that are greater than 8 µm in diameter are false colored red, while those that are equal to or less than 8 µm are false colored green. The contrast of the original panel was adjusted in (**c**) to match the SCA+CT overlay in (**d**) in order to enable direct visual comparison. Scale bar 200 µm. The diameter (**e**), total density (**f**), density of the smaller nuclei (green, (**g**)) and density of the larger nuclei (red, (**h**)) were quantified for the 3 tumor images (images 3,6, 9), 3 muscle images (images 1,4,7), and 3 tumor+muscle images (images 2,5,8). For diameter, the parenthetical values indicate the number of nuclei whose diameter were quantified. For density, the parenthetical values indicate the number of images in which the density was calculated. In (**e**) the vertical dotted red line corresponds to an 8 µm diameter, and the horizontal color bars show the mean and standard deviation for each variable.

Images captured from frozen tissue sections mounted on glass slides were used to facilitate more direct comparison of the SCA+CT to hematoxylin and eosin (H&E) stained micrographs. For this experiment, the excised tissue was frozen, sectioned serially, and mounted on glass slides. Alternating 50 µm thick and 5 µm thick sections were cut. The 50 µm thick section was imaged with the fluorescence microendoscope, and the proceeding 5 µm thick section was stained with H&E and imaged with a standard transmission microscope. This protocol was followed so that optimal images could be acquired of both the H&E stained tissue and the acriflavine stained tissue. **Fig. 4b** contains a panel of representative fluorescence microendoscopy (FM) images and the corresponding H&E *en face* section micrograph (**Fig. 4a**). The approximate locations where FM images were collected are indicated by squares in the H&E section. The tissue types in the H&E section generally correspond with the tissue types observed in the FM images; however features do not match up perfectly because these panels were acquired from different tissue slices. Nuclei in the panel of images were isolated with SCA+CT (**Fig. 4d**) as described previously. The SCA+CT panel (**Fig. 4d**) is shown as an overlay in which nuclei isolated through SCA+CT are overlaid onto the original image. Nuclei that are greater than 8 µm in diameter are false colored red, while those that are equal to or less than 8 µm are false colored green. The cutoff of ‘8 µm’ was chosen because two distinct peaks were observed in the histogram of nuclear diameters shown in **Fig. 4e** **.** Specifically, one peak was located at approximately 5 µm and the other was located at approximately 10 µm, while 8 µm appeared to be located at the dip between these two peaks. This two-color scheme is used throughout the remainder of the manuscript to highlight the bimodal distribution observed in **Fig. 4e**. The images with false colored nuclei were contrast-stretched in order to enable increased visibility of the false colored nuclei. The contrast of the original panel was adjusted in **Fig. 4c** to match the SCA+CT overlay in **Fig. 4d** in order to enable direct visual comparison. As seen, SCA+CT isolates nuclei that visually correspond to the locations of nuclei observed in the H&E micrograph (gold standard). Next, the nuclear size and density for images in the panel that contained mostly tumor tissue (3, 6, 9), mostly muscle tissue (1, 4, 7), and a mixture of tumor+muscle tissue (2, 5, 8) were quantified using SCA+CT (**Fig. 4e, f**). The size or nuclear diameter results are plotted a histograms or probability distribution functions (pdfs) to show the small differences seen in nuclear size between groups. The vertical dotted red line corresponds to the 8 µm diameter cutoff, and the horizontal color bars show the mean and standard deviation for each variable. The density results are shown are boxplots. For all boxplots shown in this work, the red line corresponds to the median and the edges of the box correspond to the 25th and 75th percentiles. The whiskers correspond to the most extreme data points not considered outliers, and outliers are plotted individually. Because there are only 3 images in each group, no statistical analysis was performed; however, a general decreasing trend in nuclear density from tumor to tumor+muscle to muscle tissue can be observed. This trend is observed for both smaller nuclei (green) and larger nuclei (red). However, there appears to be larger differences between tumor and muscle as well as tumor+muscle and muscle for the smaller nuclei (green).

### Comparison of Nuclear Size and Density in Positive and Negative Excised Tumor Margins

**Figure 5:**
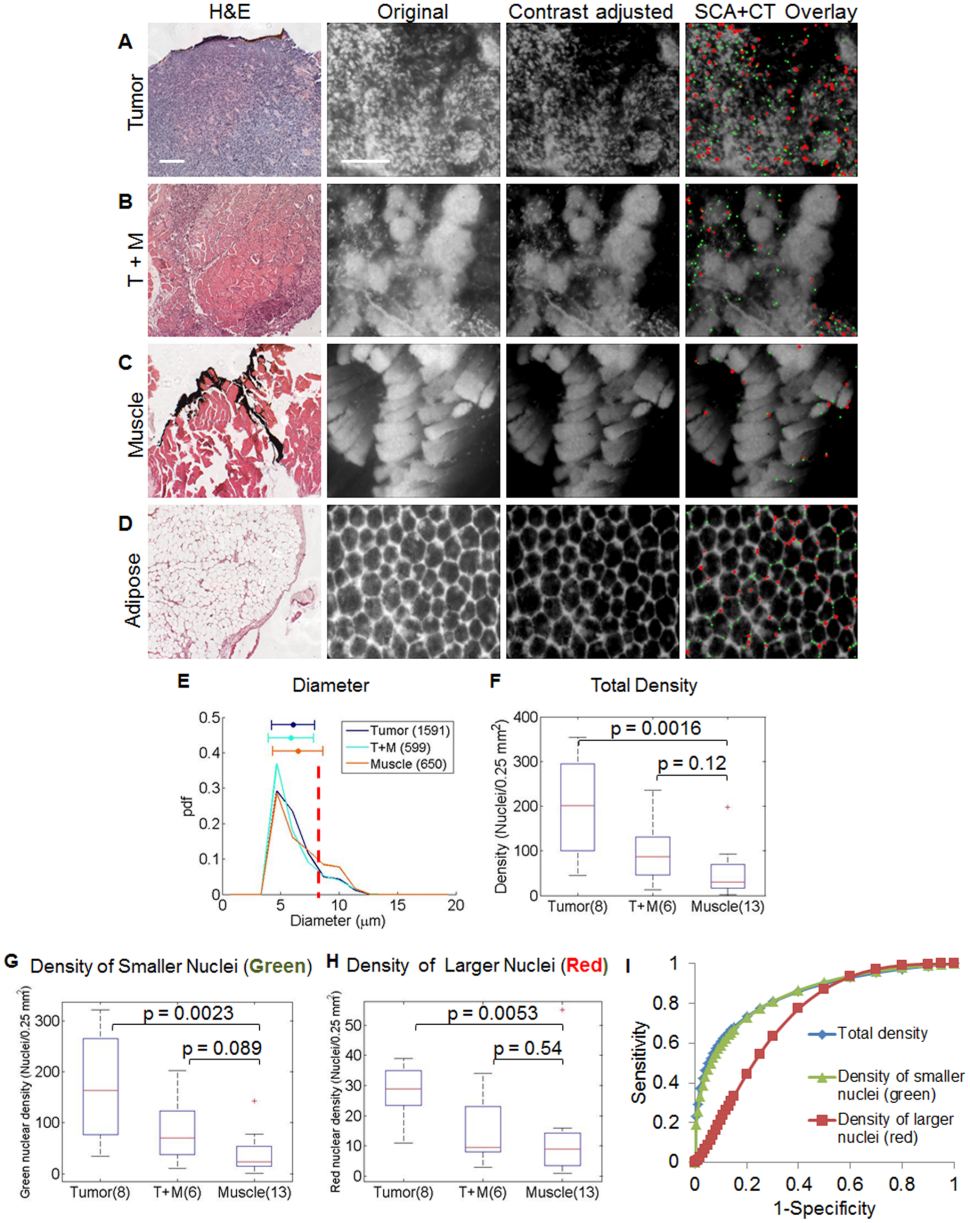
SCA+CT yields differences between *ex vivo* tissue types. Representative H&E images (column 1) of tumor, tumor+muscle (T+M), muscle, and adipose tissue and their corresponding fluorescence microendoscopy (FM) image (column 2) are shown (**a**)–(**d**) respectively. Nuclei were isolated and quantified by using SCA+CT and were overlaid onto the original image, which is illustrated in column 4. Nuclei that are greater than 8 µm in diameter are false colored red, while those that are equal to or less than 8 µm are false colored green. In column 3, the contrast of the original panel was adjusted to match the SCA+CT overlay in column 4. Scale bar 200 µm. The nuclear size (**e**), total density (**f**), density of the smaller nuclei (green, (**g**)), and density of the larger nuclei (red, (**h**)) were calculated for a cohort of 8 tumor, 6 T+M, and 13 muscle images. For diameter, the parenthetical values indicate the number of nuclei whose diameter was quantified. For density, the parenthetical values indicate the number of images in which the density was calculated. In (**e**) the vertical dotted red line corresponds to an 8 µm diameter, and the horizontal color bars show the mean and standard deviation for each variable. Significant differences are seen between tumor and muscle images for total density and the density of the smaller nuclei (green). (**i**) An ROC curve was generated using total density, density of the smaller nuclei (green), and density of the larger nuclei (red) as classifiers. The area under the curve (AUC) for each of the classifiers is 0.84, 0.84, and 0.73 respectively.

SCA+CT was applied to *ex vivo* FM images from the margins of freshly excised tumors, and representative images are shown in **Fig. 5a–d**. **Fig. 5a** contains an H&E micrograph (column 1) of tumor and the corresponding FM image (column 2). The nuclei from these images were isolated using SCA+CT, which can be seen in column 4. The images in column 4 were contrast-stretched in order to enable increased visibility of the false colored nuclei. The contrast of the original panel was adjusted in column 3 to match the SCA+CT overlay in column 4 in order to enable direct visual comparison. Additionally, the approximation, spatial, DCT, and curvelet outputs for these representative images are shown in **Fig. S2**. The SCA+CT processed tumor image shows a dense, disorganized collection of nuclei, which is characteristic of malignant tissue. **Fig. 5b–d** contain images of tumor+muscle, muscle, and adipose respectively. The SCA+CT processed tumor+muscle image contains a slightly less dense collection of nuclei than is seen in the tumor image, which is characteristic of residual tumor, while the SCA+CT processed muscle image contains few nuclei, as is characteristic of muscle or fibrous tissue. In adipose tissue, nuclei are located at the periphery of the adipocytes. In the adipose image in Figure 5D, SCA+CT correctly isolates evenly spaced nuclei that are located on the border of the adipocytes. Probability density functions and boxplots of the differences observed in nuclear size (**Fig. 5e**) and density (**Fig. 5f**) respectively were calculated for a cohort of 8 tumor, 6 tumor+muscle, and 13 muscle images. Additionally, because the combination of using both nuclear size and density could have higher diagnostic power; the density of the smaller nuclei (green) and the larger nuclei (red) were also calculated and are shown in **Fig. 5g** and **Fig. 5h** respectively. Significant differences are seen between tumor and muscle images for the total density (*p* = 0.0016), density of the smaller nuclei (green, *p* = 0.0023), and the density of the larger nuclei (red, *p* = 0.0053). The statistical differences between the tumor+muscle and muscle images are not significant for the total nuclear density, the density of the smaller nuclei (green), or the density of the larger nuclei (red); however, the difference appears the greatest for the smaller nuclei (green). Additionally, a ROC curve was generated using the total density, density of the smaller nuclei (green), and density of the larger nuclei (red) as classifiers, which resulted in an AUC of 0.844, 0.843, and 0.734 respectively (**Fig. 5i**). The sensitivity and specificity associated with the Youden index for each classifier is 73%/80%, 73%/80%, and 77%/60% respectively.

### Quantification of Nuclear Size and Density from Images of the Resected Tumor Cavity

**Figure 6:**
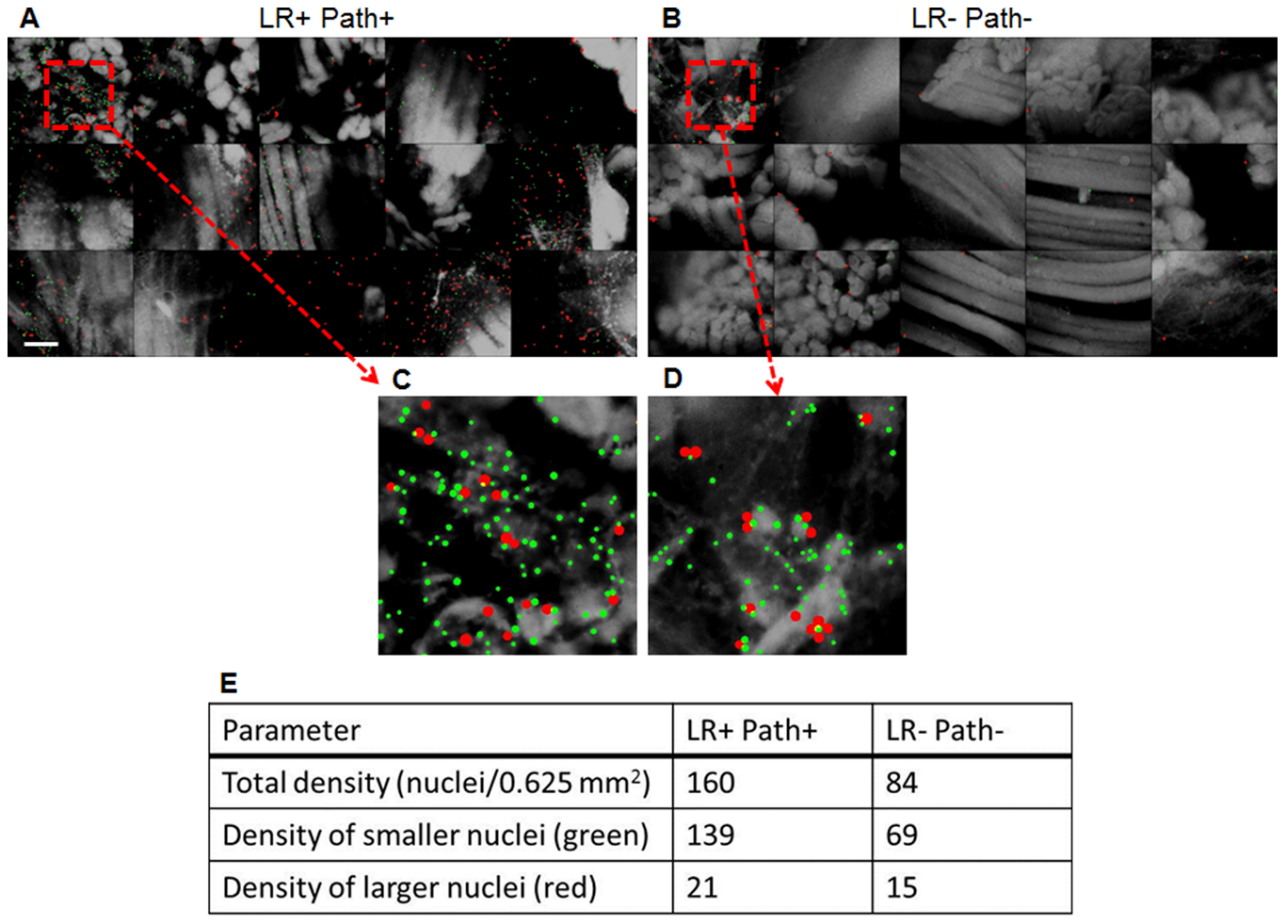
Differences observed between *in vivo* margins. Fluorescence microendoscopy (FM) image panels of a local recurrence positive (LR+)/pathology positive (Path+) (**a**) and a local recurrence negative (LR−)/pathology negative (Path−) (**c**) margin were captured *in vivo* by translating the probe over the resection cavity. The nuclei from these images were isolated with SCA+CT, and nuclei were overlaid onto the original image. Nuclei that are greater than 8 µm in diameter are false colored red, while those that are equal to or less than 8 µm are false colored green. Scale bar 200 µm. The highest density regions seen in (**a**) and (**b**) were identified and are shown in (**c**) and (**d**) respectively. The total density, density of the smaller nuclei (green), and density of the larger nuclei (red) in these regions were quantified and are shown in (**e**).

Panels of *in vivo* FM images captured from positive and negative tumor margins were analyzed with SCA+CT (**Fig. 6a–b**). In this case, two independent endpoints were measured. First, a pathological diagnosis of the excised margin was obtained, which yielded the clinical endpoint of pathologically positive (Path+) and negative (Path−) margins. Additionally, mice were monitored for local recurrence at the excision site for 120 days to distinguish between mice that recurred locally (LR+) and mice that did not recur locally (LR−). FM image panels of a LR+/Path+ and a LR−/Path− margin were captured *in vivo* by translating the probe over the resection cavity. SCA+CT is able to isolate nuclei from each panel, regardless of the presence of background. While the density of each image in the panel could be quantified and plotted, it is crucial to be sensitive to small pockets of residual disease, which are characteristic of positive tumor margins. Thus, the highest density regions in **Fig. 6a** and **Fig. 6b** were identified and are shown in **Fig. 6c** and **Fig. 6d** respectively. The total density, density of the smaller nuclei (green), and density of the larger nuclei (red) in these regions are shown in **Fig. 6e**. Large differences in the total density and density of the smaller nuclei (green) are observed between LR+/Path+ and LR−/Path− margins, while the larger nuclei (red) yields a small difference between the margins.

## Discussion

FM imaging of acriflavine stained tissue combined with an algorithm that leverages sparse component analysis and circle transform (SCA+CT) provides a rapid, non-destructive and automated strategy for quantitative pathology of thick tissues with non-uniform background heterogeneity. Unlike nuclear stains such as DAPI, acriflavine stains connective tissue, such as skeletal muscle and the outline of adipose cells, in addition to nuclei. Thus in addition to being able to detect increased nuclear size and density, which are traditional hallmarks of carcinogenesis, it is also possible to place nuclei within the context of the entire histological landscape, which is illustrated in the spatial curvelet overlay in **Fig. 1** [44], [45]. The nuclei in the adipose region of the image are spatially co-registered with the adipocyte outlines captured in the curvelet bin. This information could be used to indicate whether a nucleus is associated with benign tissue such as adipose or with malignant tissue. While the focus of this manuscript was to systematically evaluate the potential of SCA+CT to quantify nuclear density for tumor margin assessment, there is the potential to incorporate the context information illustrated in **Fig. 1** in future work. This combination of approaches provides a powerful alternative to complicated and time-intensive immunohistochemistry techniques, which require fixing, sectioning, and staining and which can only be performed on the excised margin. Further, the ability to implement this technology to detect residual disease in the tumor cavity (*in vivo*) is likely to be more predictive of local recurrence.

SCA+CT achieved the lowest errors for higher contrast ratios and larger nuclear sizes. **Fig. 3** reveals that circle transform (CT) may have a lower limit to the size of nuclei that it is able to detect. More specifically, CT has difficulty identifying nuclei whose diameter is less than 5 pixels. While tissue simulations provided insight into the performance of SCA+CT and how it varies with nuclear size, density, and contrast, we did not include variation in the shape of our nuclei, which could bias the output of the circle transform. Additionally, SCA+CT correctly isolates nuclei in *ex vivo* images and shows consistently increased density in tumor and tumor+muscle images compared to images containing muscle (**Fig. 4** **,** **5**). Furthermore, slightly larger differences in density were seen between tumor+muscle and muscle images in the smaller range of nuclear size (<8 µm diameter, green nuclei), suggesting that using a combination of nuclear size and density information may achieve higher contrast between tumor+muscle and muscle tissue images. For both the total density and density of the smaller nuclei (green) in **Fig. 5**, the AUC was approximately 0.84 and the corresponding sensitivity and specificity were 73% and 80% respectively. It is difficult to comment on the clinical acceptability of the performance of our method for several reasons. First, the 73% and 80% sensitivity and specificity is an estimate of our methods performance when applied to distinguish between a small cohort of positive and negative images acquired from a preclinical model. To achieve a better estimate, we plan to image and analyze a larger cohort of preclinical sarcoma margins as part of our future efforts. Second, at this time intraoperative techniques for margin assessment, such as intraoperative frozen sectioning, are not widely used or accepted across clinics or institutions. Thus, it is difficult to compare our method to another clinical method. Standard of care includes visual inspection of the lesion by the surgeon and post-operative histopathology of the excised margin. In soft-tissue sarcoma, although reported rates of positive margins range from 10–18%, the local recurrence rates are higher (13–31%), which could in part be due to incomplete sampling of the large tissue samples in post-operative pathology, which underestimates the true positive margin rate and the potential influence of adjuvant radiation therapy [46], [47]. Lastly, when used to image and quantify the micro histology of the resection cavity, SCA+CT shows higher nuclear density in the LR+/Path+ margin than the LR−/Path− margin (**Fig. 6**). The ability to implement this technology directly within the resection cavity allows for the detection of residual disease directly in the cavity rather than inferring indirectly from the excised tumor margin, which may be more predictive of eventual recurrence probability.

While SCA+CT is optimized in a sarcoma margin model with a fluorescence microendoscope, the proposed strategy for performing *in situ* quantitative pathology lends itself easily to other types of morphological imaging. Specifically, in this manuscript the impact of contrast is investigated in **Fig. 3** to essentially emulate different contrast ranges that would be true of different microscopy techniques. SCA+CT can isolate nuclei even at ratios of 1.2 (max tumor nuclei intensity/max background intensity). As mentioned previously, **Fig. 3** reveals that circle transform (CT) may have a lower limit to the size of nuclei that it is able to detect (less than 5 pixels). While this is potentially beneficial in that CT will not pick up small artifacts or aberrations, it could also miss nuclei if the pixel size is too large (and the resolution is too low). Taken together, the analytical methodology described here may be generalized to different imaging techniques and staining approaches as long as certain conditions are met. Specifically, conditions include situations in which images have components or sources that can be represented sparsely in a given basis (for example nuclei in the pixel basis, muscle in a frequency basis) and the elements of one basis cannot be sparsely represented in any of the other bases. Additionally, while standard Gaussian noise models are assumed in the development of the SCA+CT algorithm, the fundamental ideas can be easily extended to photon noise models common in fluorescence, confocal, and multiphoton microscopy [33], [48]–[50].

In this study we did not focus on adipose tissue as our data set primarily contained tumor and muscle tissues, which is characteristic of sarcoma. Moving forward we plan to focus more on adipose tissue, which may play a larger role in other heterogeneous cancers, such as breast cancer, and explore how the information in it may be used diagnostically. Additionally, we did not focus on nuclear size quantification because our ability to delineate small variations in nuclear size is limited by the resolution of the fluorescence microendoscope. However, nuclear size may have significant diagnostic value when examined in images that were captured with a system that has higher resolution.

Fluorescence imaging of tissue micro-anatomy combined with a specialized algorithm for delineation and quantification of nuclear features is a means for rapid, non-destructive and automated detection of microscopic residual disease in surgical tumor margins. This strategy has the potential to be extended to other tumors and organ sites with significant and non-uniform background heterogeneity, can be seamlessly translated to the *in vivo* setting, and ultimately may provide valuable quantitative information to surgeons during a procedure.

## Supporting Information

Figure S1 **Image preprocessing.** The original image of a tumor+muscle site is shown in (**a**). (**b**) The image is first cropped to remove the rim of the fiber bundle. (**c**) Next a low pass Gaussian filter is applied to remove the fiber cores that are superimposed on the image. The image displayed in (**c**) is the input into the sparse decomposition algorithm.
(TIF)

Figure S2 **SCA outputs from** ***ex vivo*** **tissue types.** (**a**)–(**d**) Representative high resolution images (column 1) of tumor, tumor+muscle, muscle, and adipose tissue are shown in rows 1–4 respectively. The approximation, spatial, DCT, and curvelet outputs are shown in columns 2–5 respectively.
(TIF)

Methods S1 **Description of additional methods.**
(DOCX)
