# Supplementary figures and images for: Quantitative Segmentation of Fluorescence Microscopy Images of Heterogeneous Tissue: Application to the Detection of Residual Disease in Tumor Margins
Source: PLoS One. 2013 Jun 18;8(6):e66198. doi: 10.1371/journal.pone.0066198 (PMC3688889; doi:10.1371/journal.pone.0066198)

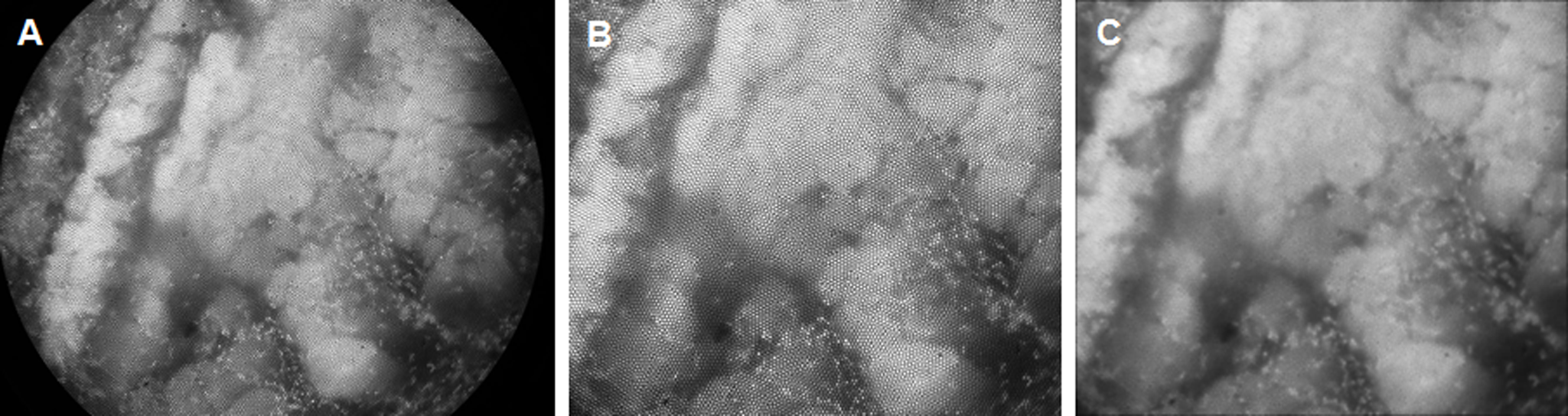

Supplement: Figure S1 — Image preprocessing. The original image of a tumor+muscle site is shown in (a). (b) The image is first cropped to remove the rim of the fiber bundle. (c) Next a low pass Gaussian filter is applied to remove the fiber cores that are superimposed on the image. The image displayed in (c) is the input into the sparse decomposition algorithm. (TIF) [file pone.0066198.s001.tif]

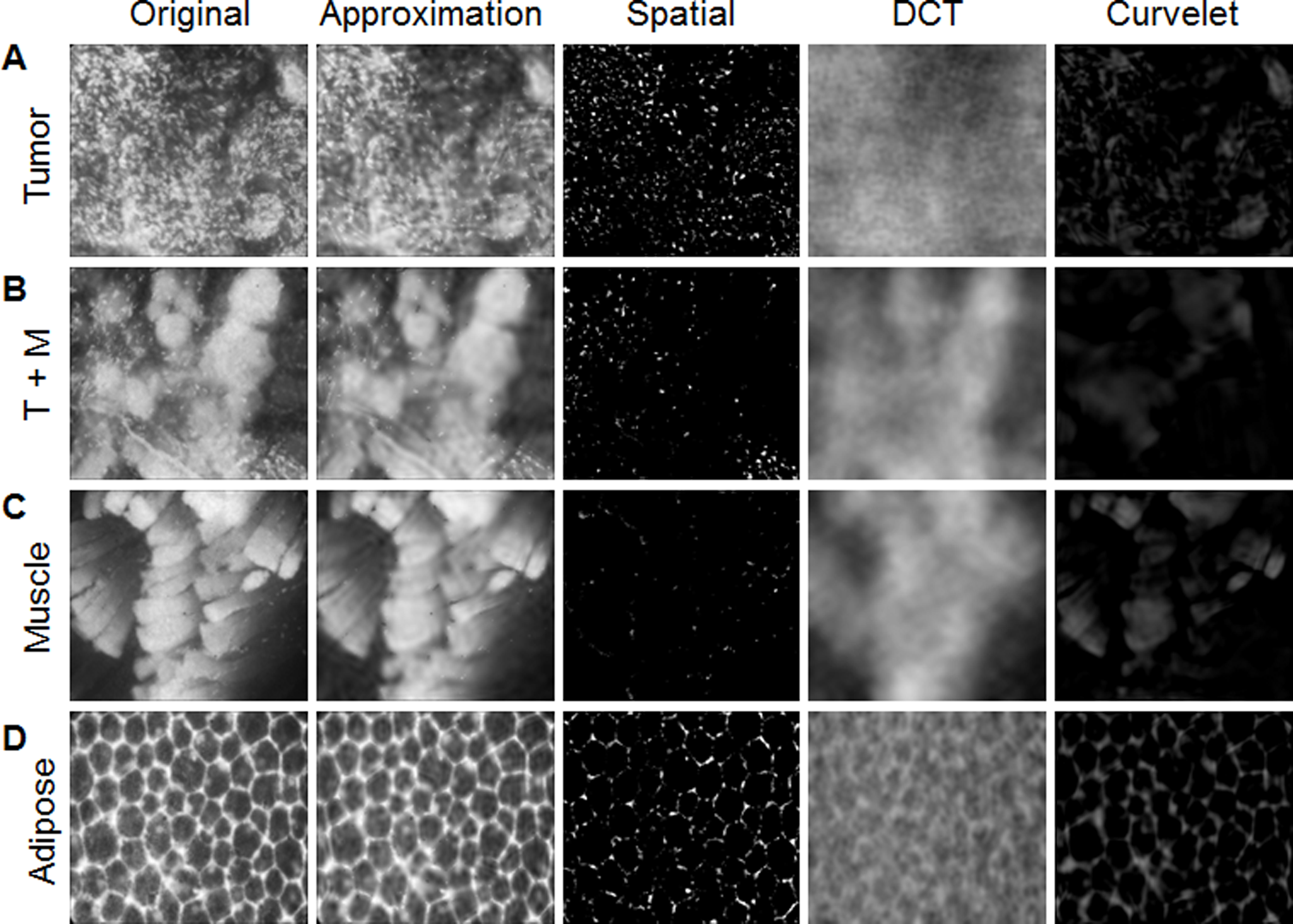

Supplement: Figure S2 — SCA outputs from ex vivo tissue types. (a)–(d) Representative high resolution images (column 1) of tumor, tumor+muscle, muscle, and adipose tissue are shown in rows 1–4 respectively. The approximation, spatial, DCT, and curvelet outputs are shown in columns 2–5 respectively. (TIF) [file pone.0066198.s002.tif]
