# Supplementary material for: Quantitative Segmentation of Fluorescence Microscopy Images of Heterogeneous Tissue: Application to the Detection of Residual Disease in Tumor Margins
Source: PLoS One. 2013 Jun 18;8(6):e66198. doi: 10.1371/journal.pone.0066198 (PMC3688889; doi:10.1371/journal.pone.0066198)
Supplement: Methods S1 — Description of additional methods. (DOCX) [file pone.0066198.s003.docx]

***Tissue simulations***

To select a biologically appropriate range of values for the nuclear sizes in the tumor simulation, a pathologist was consulted. Typically, nuclei in a sarcoma mouse model range from 5 - 15 µm. Therefore, to fully cover the biologically observed range tumor nuclei expected with our system (in which there were 1.36 pixels per µm), nuclei were simulated with diameters that ranged from 4 up to 18 pixels (3 to 13 µm). Densities were simulated from 60-900 nuclei/ 0.25 mm^2^. This range was selected to ensure that a single nucleus could be detected (at the lowest end) all the way up to a dense collection of nuclei in which all nuclei were touching (at the highest end). For the tumor + muscle simulations, the ratios between tumor nuclei and the underlying muscle were chosen based on observations from experimental images and were varied from 1.2-1.8 (max tumor nuclei intensity/ max muscle intensity).

***Image preprocessing***

Prior to applying the SCA to the acquired images, the images were preprocessed to remove the rim of the fiber bundle and the fiber core pattern superimposed onto each image. Images were cropped to retain only the central portion containing an image of the tissue sample (**Fig S1b**). Next, a low-pass Gaussian filter with a full-width half-maximum (FWHM) size of 4.7 µm was applied to remove the high-frequency fiber bundle pattern (**Fig S1c**). This FWHM was selected such that it approximately matched the bandwidth of the Nyquist cutoff frequency for the fiber bundle pattern, equal to half of the distance between fiber cores [51].

***Regularization parameter selection***

Four representative experimental images (one tumor, one muscle, one adipose, and one tumor + muscle image) were used to systematically select the best set of regularization parameters. Each image was analyzed with sixty-four different combinations of $\tau_{\text{nuclei}}$, $\tau_{\text{muscle}}$, and $\tau_{\text{adipose}}$. For each triplet of parameters, a series of questions were asked of two independent reviewers: (1) does $\hat{x}_{\text{nuclei}}$ capture the nuclei present across the 4 representative images, (2) does $\hat{x}_{\text{adipose}}$ capture the curved features in the 4 images (such as the outline of adipose cells), (3) does $\hat{x}_{\text{muscle}}$ capture the low frequency features in the 4 images (such as the periodic longitudinal muscle fibers)? The reviewers determined that parameters $\tau_{\text{nuclei}}=0.10$, $\tau_{\text{muscle}}=1.0$, and $\tau_{\text{adipose}}=1.0$ gave strong performance across tissue types; this set was applied to all images in this study.

51. Bozinovic N, Ventalon C, Ford T, Mertz J (2008) Fluorescence endomicroscopy with structured illumination. Opt Express 16: 8016-8025.
